# *ALU* transposition induces familial hypertrophic cardiomyopathy

**DOI:** 10.1002/mgg3.951

**Published:** 2019-09-30

**Authors:** Landry Nfonsam, Lijia Huang, Nancy Carson, Jean McGowan‐Jordan, Melanie Beaulieu Bergeron, Sharan Goobie, Susan Conacher, David McCarty, Lee Benson, Stacy Hewson, Laura Zahavich, Elizabeth Sinclair‐Bourque, Amanda Smith, Ryan Potter, Mahdi Ghani, Lucas Bronicki, Olga Jarinova

**Affiliations:** ^1^ CHEO Ottawa Canada; ^2^ University of Ottawa Ottawa Canada; ^3^ Dalhousie University Halifax Canada; ^4^ London Health Sciences Centre London Canada; ^5^ Western University London Canada; ^6^ The Hospital for Sick Children Toronto Canada

**Keywords:** cardiomyopathy, copy number, deletion, MYBPC3

## Abstract

**Background:**

Hypertrophic cardiomyopathy (HCM) is characterized by left ventricular hypertrophy (LVH) in the absence of predisposing cardiovascular conditions. Pathogenic variants in at least 16 cardiac sarcomeric genes have been implicated in HCM, most of which act in a dominant‐negative fashion. However loss‐of‐function (haploinsufficiency) is the most common disease mechanism for pathogenic variants in *MYBPC3*, suggesting that *MYBPC3* complete deletion may play a role in HCM pathogenesis. Here, we investigate *MYBPC3* complete deletion as a disease mechanism in HCM by analyzing two unrelated patients with confirmed diagnosis of HCM that tested negative by Sanger sequencing analysis.

**Methods:**

*MYBPC3* complete deletion was investigated by Multiplex ligation‐dependent probe amplification (MLPA) and microarray analyses. The mechanism of deletion was investigated by interrogating the SINEBase database.

**Results:**

Patient‐1 was diagnosed with nonobstructive HCM in his mid‐40s while undergoing assessment for palpitations, and patient‐2 with obstructive HCM in his late‐20s while undergoing systolic heart murmur assessment for an unrelated illness. MLPA testing revealed a heterozygous deletion of all *MYBPC3* exons in both patients. Subsequent microarray testing confirmed these deletions which extended beyond the 5′ and 3′ ends of *MYBPC3*. Genomic assessment suggested that these deletions resulted from *Alu/Alu*‐homologous recombination.

**Conclusion:**

Our results demonstrate that haploinsufficiency resulting from *MYBPC3* complete deletion, potentially mediated by *Alu* recombination, is an important disease mechanism in cardiomyopathy and emphasizes the importance of copy number variation analysis in patients clinically suspected of HCM.

## Graphical abstract

We report on the complete deletion of MYBPC3, potentially mediated through Alu recombination, in two patients with confirmed diagnosis of hypertrophic cardiomyopathy (HCM). Our result demonstrates for the first time that MYBPC3 complete deletion is an important disease mechanism in cardiomyopathy and emphasizes the importance of copy number variation analysis in patients clinically suspected of HCM.

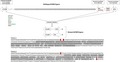

## 1 INTRODUCTION

Hypertrophic cardiomyopathy (HCM) is characterized by the presence of left ventricular hypertrophy (LVH) in the absence of predisposing cardiovascular or systemic conditions (Cirino & Ho, 2008). HCM is inherited in an autosomal‐dominant manner and pathogenic variants in at least 16 genes, the majority of which are missense that act in a dominant‐negative manner, have been implicated (Cirino & Ho, 2008). One of the principal genes in HCM is myosin‐binding protein C, cardiac (*MYBPC3*: OMIM_600958), which plays a structural role and modulates myocardial contraction. About 70% of pathogenic variants in *MYBPC3* are nonsense/frameshift (Carrier, Schlossarek, Willis, & Eschenhagen, 2010) suggesting haploinsufficiency as the pathogenic mechanism for *MYBPC3*. Although partial deletions in *MYBPC3* have been implicated in HCM (Chanavat et al., 2012; Lopes et al., 2015), a complete deletion has not been reported before. We present two HCM patients with a complete deletion of *MYBPC3* and investigated the potential origin of these deletions.

## 2 METHODS

### 2.1 Ethical compliance

All procedures followed were in accordance with the Helsinki Declaration of 1975, as revised in 2000. Informed consent was obtained from all patients in accordance with routine ethical and clinical standards. Additional informed consent was obtained for identifying information included in this article.

### 2.2 Sample collection and multiplex ligation‐dependent probe amplification

Multiplex ligation‐dependent probe amplification (MLPA) analysis using the SALSA P100‐B1 (patient‐1) or P100‐MYBPC3 (patient‐2) probe kits (MRC‐Holland, Amsterdam, the Netherlands) was performed as previously described (Chanavat et al., 2012) on 1622 individuals with clinical diagnosis or suspicion of inherited HCM referred to our laboratory between January 2014 and December 2017. Results were analyzed using the Coffalyser software v.140429.1057 (patient‐1) or v.140721.1958 (patient‐2) (MRC‐Holland).

### 2.3 Microarray and genome sequence analysis

Microarray analysis using CytoscanHD (ThermoFisher Scientific) was performed following standard procedures as previously described (Palumbo et al., 2014), and data were analyzed with the Chromosome Analysis Suite software (ThermoFisher Scientific) using the GRCh37/hg19 genome assembly. Sequence analysis for recombination repeat elements was conducted by interrogating the SINEBase database v.1.1 (http://sines.eimb.ru/) (Vassetzky & Kramerov, 2013).

### 2.4 GenBank reference and version numbers for HCM‐Tested Genes

MYBPC3 (NG_007667.1), MYH7 (NG_007884.1), TNNT2 (NG_007556.1), TNNI3 (NG_007866.2), TPM1 (NG_007557.1).

## 3 RESULTS AND DISCUSSION

Patient‐1 is a 64‐year‐old male who was diagnosed with nonobstructive HCM at age 46 (Figure S1). His echocardiogram revealed moderately dilated left and right atria, and magnetic resonance imaging showed severe asymmetric LVH and interventricular septal wall thickness. Electrocardiogram (ECG) revealed atrial flutter and ST‐T wave abnormalities (Figure S2).

**Figure 1:**
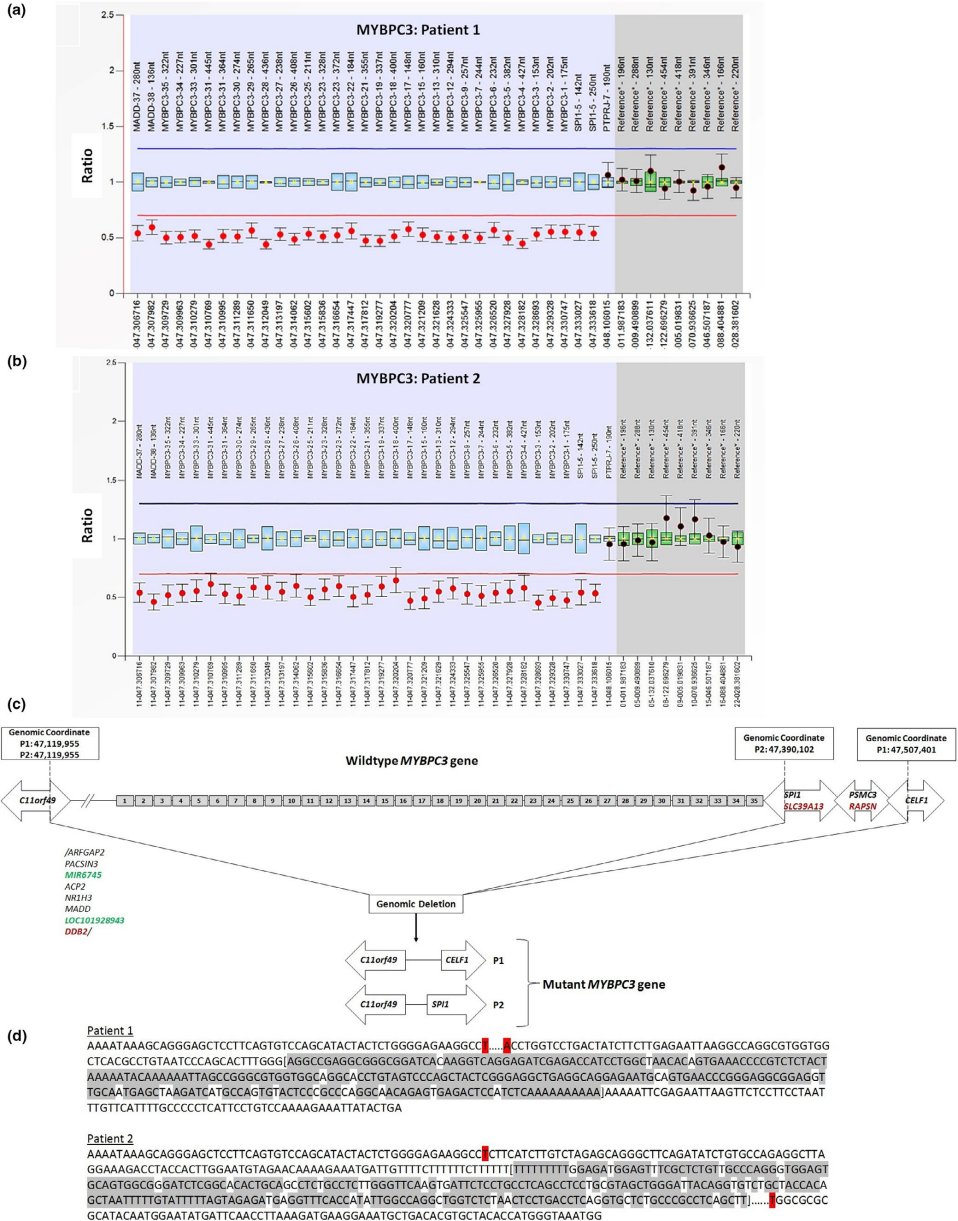
(a and b) MLPA analysis of *MYBPC3* for patient‐1 (P1) and patient‐2 (P2). Probe ratio shows deletion spanning *MYBPC3* and extending into *MADD* and *SPI1*. (c) Illustration of wild‐type *MYBPC3*, and the microarray‐detected heterozygous deletions: 387 kb deletion spanning *MYBPC3* and 10 OMIM‐genes (patient‐1); 270 kb deletion spanning *MYBPC3* and 6 OMIM‐genes (patient‐2). Highlights: OMIM‐morbid genes → red; RNA‐genes → green. (d) *Alu*‐mediated deletion. *Alu*(5′)‐*Alu*(3′) common sequences → in brackets; Nucleotide homology to *Alu* sequence → grey_highlight; Recombination‐window/breakpoints → red_highlight. GenBank reference and version numbers for HCM Tested Genes: *MYBPC3* (NG_007667.1), MYH7 (NG_007884.1), TNNT2 (NG_007556.1), TNNI3 (NG_007866.2), and TPM1 (NG_007557.1). HCM, hypertrophic cardiomyopathy; MLPA, multiplex ligation‐dependent probe amplification

Sanger sequencing (SS) of the five genes most commonly associated with HCM [*MYBPC3*, myosin heavy chain 7 (*MYH7*: OMIM_160760), troponin T2, cardiac type (*TNNT2*: OMIM_191045), troponin I3, cardiac type (*TNNI3*: OMIM_191044), and tropomyosin 1 (*TPM1*: OMIM_191010)] was negative. Analysis for large deletions and/or duplications by MLPA showed that all exons of *MYBPC3* and part of MAP kinase activating death domain (*MADD*: OMIM_603584) and Spi‐1 proto‐oncogene (*SPI1*: OMIM_165170), located 5′ and 3′ of *MYBPC3*, respectively, were present in single copies. This indicated a large deletion that includes the entire *MYBPC3* (Figure 1a). Microarray analysis was performed to confirm and delineate the deletion breakpoints. Results showed a heterozygous deletion of 387 kb on chromosome 11p11.2 (arr[GRCh37]:47119955_47507401) that encompassed *MYBPC3* and three other OMIM‐morbid genes: damage‐specific DNA‐binding protein 2 (*DDB2*: OMIM_600811), solute carrier family 39 member 13 (*SLC39A13*: OMIM_608735), and receptor‐associated protein of the synapse (*RAPSN*: OMIM: 601,592) (Figure 1c). Pathogenic variants in *DDB2*, *SLC39A13*, and *RAPSN* cause autosomal recessive disorders (OMIM, 2019), and based on evidence to date, the heterozygous deletion of *DDB2*, *SLC39A13*, and *RAPSN* is not expected to have clinical consequences in this patient.

Patient‐2 is a 46‐year‐old male with no known family history of cardiomyopathy, who was diagnosed with obstructive HCM characterized by marked LVH at age 27 (Figure S1). His ECG revealed sinus bradycardia and secondary ST‐T changes (Figure S2). Previous sequencing tests yielded negative results. However, MLPA analysis showed complete deletion of *MYBPC3* (Figure 1b), and microarray analysis revealed a 270 kb deletion on chromosome 11p11.2 (arr[GRCh37]: 47119955_47390102) encompassing *MYBPC3*, *DDB2*, and *SPI1* 3′‐end (Figure 1c).

To determine if the overlapping deletions in patient‐1 and 2 were mediated by homologous recombination, short‐interspersed‐nuclear‐element (SINE) analysis (http://sines.eimb.ru/) (Vassetzky & Kramerov, 2013) of sequences adjacent to the deletion breakpoints was performed. Results revealed a 290bp *Alu* sequence located downstream in intron‐2 of chromosome 11 open reading frame 49 (*C11orf49*), and another upstream in intron‐6 of CUGBP Elav‐like family member 1 (*CELF1*: OMIM_601074) (patient‐1) or intron‐2 of *SPI1* (patient‐2) (Figure 1d). This suggests that, previously reported homologous recombination between *Alu* sequences (Chanavat et al., 2012) is likely the mechanism for deletions in these patients.

To our knowledge, this is the first report of patients where a complete deletion of *MYBPC3* has contributed to the development of HCM. Despite recent reports of the use of high‐throughput next‐generation sequencing for copy number variation (CNV) analysis (Lopes et al., 2015), because this approach had not yet been optimized in our laboratory, deletions were detected by MLPA and confirmed by microarray. Our data suggest that *MYBPC3* complete deletion, potentially mediated by *Alu* recombination is an important disease mechanism in cardiomyopathy and emphasize the importance of CNV analysis in patients clinically suspected of HCM, especially patients who tested negative by SS or other methods not optimized for CNV detection.

## CONFLICT OF INTEREST

None.

## Data Availability

Available from the corresponding author upon reasonable request.
