# Supplementary figures and images for: ALU transposition induces familial hypertrophic cardiomyopathy
Source: Mol Genet Genomic Med. 2019 Sep 30;8(1):e951. doi: 10.1002/mgg3.951 (PMC6978237; doi:10.1002/mgg3.951)

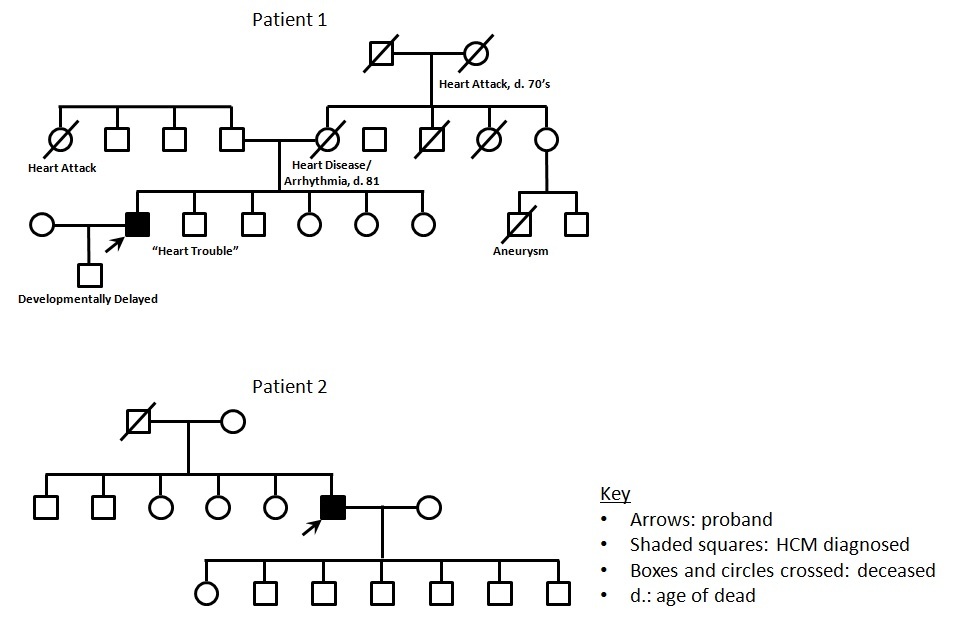

Supplement: Supplementary file 1 [file MGG3-8-e951-s001.jpg]

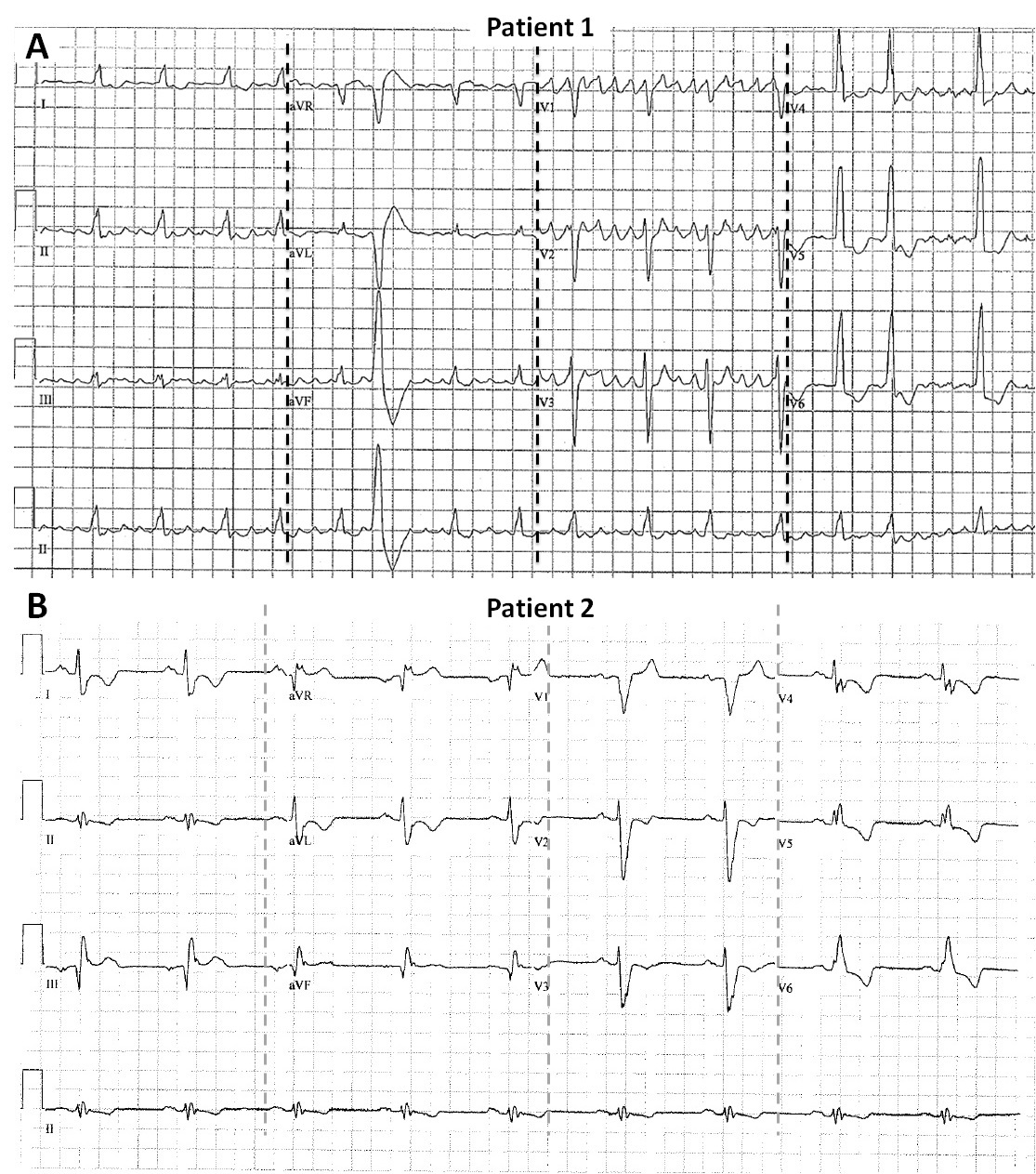

Supplement: Supplementary file 2 [file MGG3-8-e951-s002.jpg]
